# Research progress on the role of gut microbiota dysbiosis in the pathogenesis of immune−mediated liver diseases

**DOI:** 10.3389/fimmu.2026.1708826

**Published:** 2026-03-09

**Authors:** Yang Chen, Zhenglian Wang, Yixuan Zeng, Xuqiu Xie, Linquan Liu, Yan Qian

**Affiliations:** 1 Department of Emergency, Shenzhen Longhua District Central Hospital, Shenzhen, China; 2 Department of Emergency, Changsha Hospital of Traditional Chinese Medicine, Changsha, China; 3 Medical College, Jiaying University, Meizhou, China; 4 Office of the Dean, Shenzhen Longhua District Central Hospital, Shenzhen, China; 5 Department of Chronic Disease Management, The First Hospital of Hunan University of Chinese Medicine, Changsha, China; 6 Public Relations Section, Shenzhen Longhua District Central Hospital, Shenzhen, China

**Keywords:** epidemiology, gut microbiota dysbiosis, immune−mediated liver diseases, immunomodulation, therapeutic management

## Abstract

Gut microbiota dysbiosis plays a significant role in the pathogenesis of immune-mediated liver diseases (IMLDs), including autoimmune hepatitis (AIH), primary biliary cholangitis (PBC), and primary sclerosing cholangitis (PSC), through multiple gut-liver axis mechanisms. Microbial metabolites such as short-chain fatty acids (SCFAs) and secondary bile acids regulate hepatic immune homeostasis by activating G protein-coupled receptors (GPRs) and the farnesoid X receptor (FXR). Concurrently, disruption of the intestinal barrier integrity allows endotoxins (e.g., lipopolysaccharide) to activate hepatic macrophages via the TLR4/NF-κB pathway, triggering a pro-inflammatory cytokine cascade. Studies indicate an enrichment of *Veillonella* in AIH patients, while PBC patients display elevated *Enterobacteriaceae* and reduced *Oscillospira* spp. PSC is characterized by *Klebsiella pneumoniae* translocation and *Candida albicans* toxin-mediated injury. Therapeutic strategies such as fecal microbiota transplantation (FMT), probiotics, prebiotics, and bacteriophages therapy have shown efficacy in clinical settings, underscoring the potential of targeting the gut microbiota for managing IMLDs. Future research should integrate immune cell regulation by gut-derived factors and develop precision therapies based on the gut-liver axis.

## 1 Introduction

The advent of advanced metagenomic technologies has transformed our understanding of the complex ecosystem within the human gut, which harbors over 100 trillion microorganisms with a collective gene count approximately 150 times that of the human genome (1). This vast microbial community actively regulates host physiology through the secretion of an array of bioactive molecules, including short-chain fatty acids (SCFAs), secondary bile acids (SBAs), and various immunomodulatory compounds – collectively earning recognition as a veritable “forgotten organ” (2). Notably, these gut-derived microbes possess enzymatic systems capable of performing biochemical transformations inaccessible to the host alone, fundamentally reshaping our comprehension of human-microbial symbiosis (3).

The liver serves as a critical immunological and metabolic interface, continuously processing portal venous blood that carries gut-derived antigens and microbial metabolites. Kupffer cells (KCs), the resident macrophages of the liver, act as a frontline “microbial filter” (4, 5) and contribute to establishing a tolerogenic environment that selectively monitors intestinal content. Disruption of this finely tuned gut-liver communication is increasingly viewed as an important contributor to the onset and progression of immune-mediated liver diseases (IMLDs) (6–8), such as autoimmune hepatitis (AIH), primary biliary cholangitis (PBC), and primary sclerosing cholangitis (PSC). Furthermore, dysbiosis can compromise intestinal barrier integrity (9), facilitating the translocation of bacteria and their products, which may initiate or exacerbate inflammatory liver damage (10–12). Emerging therapeutic strategies that focus on the gut-liver axis (13), including fecal microbiota transplantation (FMT) (14), phage therapy, and probiotics (15), show promise in managing related liver disorders (16).

Despite these significant advances, critical knowledge gaps persist. Current research often describes associative links between microbial shifts and disease states, but the precise causal mechanisms—defining which specific microbial taxa or metabolites drive pathology in which IMLD through which exact molecular and immunological pathways—remain inadequately elucidated. Furthermore, the comparative analysis of gut-liver axis dysregulation across the spectrum of IMLDs (AIH, PBC, PSC) is limited, hindering the development of disease-specific, microbiota-based diagnostics and therapies. There is also a translational gap between promising preclinical findings and robust clinical evidence supporting targeted interventions.

To address these gaps, this review synthesizes contemporary evidence on the interplay between gut microbiota and hepatic immune regulation. We specifically focus on dissecting the mechanistic pathways through which microbial dysbiosis contributes to the initiation and progression of AIH, PBC, and PSC. By critically evaluating the existing literature and highlighting unresolved questions, this work aims to establish a refined framework for understanding disease pathogenesis and to identify concrete avenues for developing novel, microbiota-targeted therapeutic strategies.

## 2 Gut microbiota dysbiosis in the pathogenesis of IMLDs

### 2.1 Molecular mechanisms of key gut microbiota metabolites and their regulation of IMLDs

**Figure 1:**
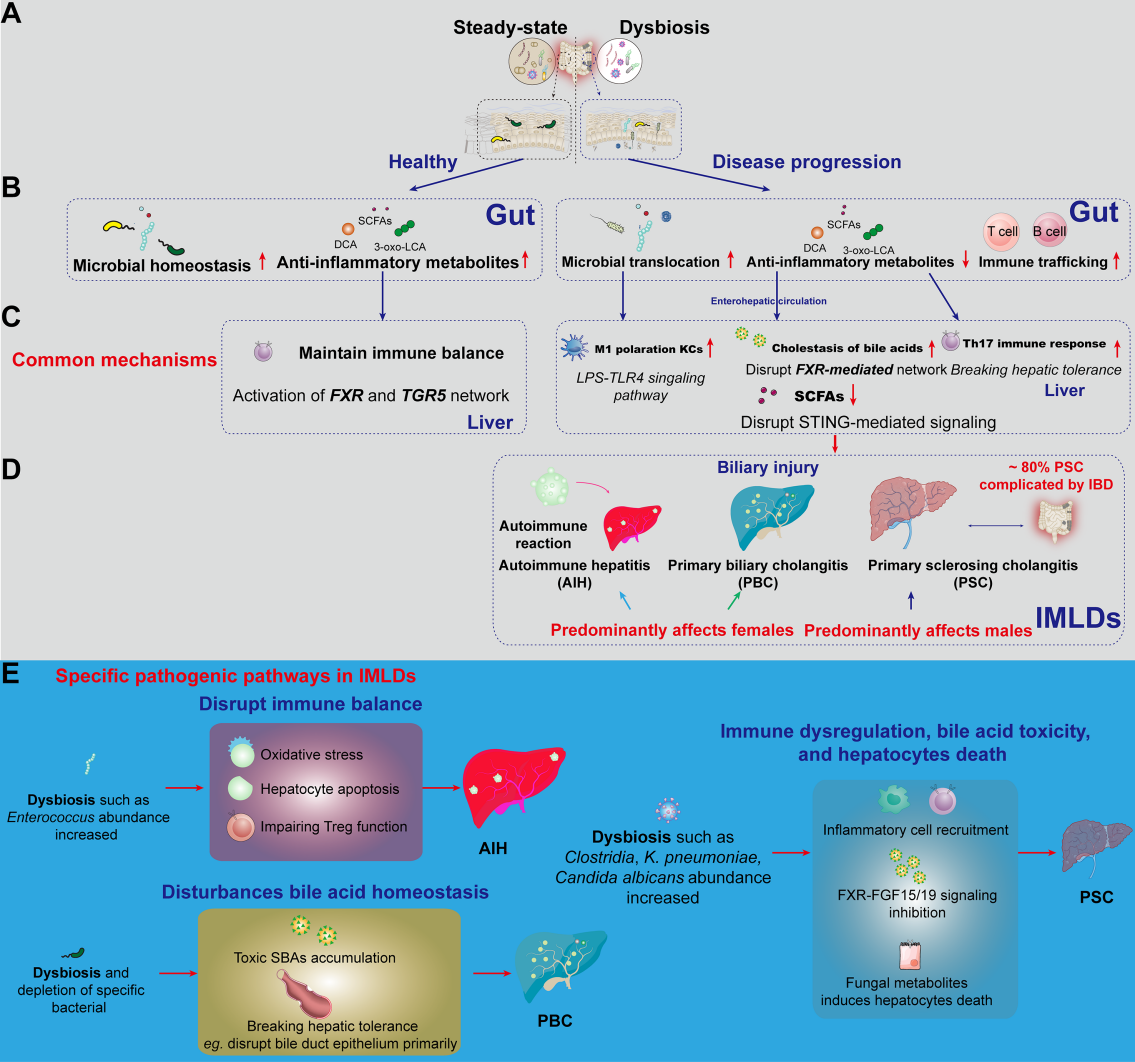
Schematic diagram of hepatic immune disease pathogenesis and epidemiological patterns induced by gut dysbiosis. **(A)** Comparison of gut homeostasis and dysbiosis. Steady-state (Left): Normal gut microbiota with stable structure (e.g., *Bacteroidetes*, *Firmicutes*), balanced host immunity. Dysbiosis (Right): Microbial dysbiosis with overgrowth of pathogenic bacteria (e.g., *Enterobacteriaceae*), damaged epithelial cells, infiltration of neutrophils/macrophages, and impaired intestinal barrier. **(B)** The underlying causes of disease progression driven by gut microbiota dysbiosis include microbial translocation, reduced levels of anti-inflammatory metabolites, and increased trafficking of immune cells. **(C)** Representative mechanisms by which metabolites from gut dysbiosis exert effects on liver immune regulation via the enterohepatic circulation. LPS- TLR4 signaling activates KCs polarize to M1 phenotype; Disrupting the FXR-mediated bile acid network; Leading to cholestasis and loss of hepatic immune tolerance. **(D)** Epidemiological characteristics of IMLDs triggered by different immune responses induced by gut metabolites. Notable epidemiological features are highlighted in red. **(E)** Specific pathogenic pathways in IMLDs. AIH: Dysbiosis (*e.g.*, increased *Enterococcus* abundance) disrupts immune balance via oxidative stress, hepatic apoptosis, and impaired Treg function. PBC: Dysbiosis (*e.g.*, increased *Clostridia*, *K. pneumoniae*, and *Candida albicans* abundance) disturbs bile acid homeostasis, leading to toxic secondary bile acids accumulation and primary disruption of bile duct epithelium. PSC: Inflammatory cell recruitment, FXR-FGF15/19 signaling inhibition, and fungal metabolites induce hepatocyte death, leading to PSC. AIH, autoimmune hepatitis; PBC, primary biliary cholangitis; PSC, primary sclerosing cholangitis; LPS, Lipopolysaccharide; TLR4, Toll-like receptor 4; IMLDs, the Immune−mediated liver diseases; SCFAs, Short-chain fatty acids; SBAs, Secondary bile acids; DCA, Deoxycholic acid; KCs, Kupffer cells; FXR, Farnesoid X receptor; FGF15/19, Fibroblast growth factor 15/19.

The gut-liver axis constitutes a highly specialized bidirectional network in which gut microbiota and their metabolites—including SCFAs and SBAs — are transported directly to the liver via the portal vein (17). This anatomical pathway enables continuous communication between intestinal microbial communities and hepatic immune cells (18), a process essential for maintaining immune tolerance and systemic homeostasis (17). Dysbiosis, characterized by microbial imbalance, disrupts this dialogue and contributes to the initiation and progression of IMLDs (**Figure 1A**). The process begins when gut-derived antigens are recognized by hepatic cells (11) such as Kupffer cells (KCs), liver sinusoidal endothelial cells (19), and various lymphocytes, which express pattern recognition receptors capable of detecting pathogen-associated molecular patterns and damage-associated molecular patterns.

Under homeostatic conditions, SCFAs and SBAs serve as potent immunomodulators (20, 21). SCFAs, primarily acetate, propionate, and butyrate, exert anti-inflammatory effects through multiple avenues: they activate G protein-coupled receptors (FFAR2/FFAR3) (22) to promote M2 macrophage polarization (23) and inhibit the NF-κB pathway, enhance intestinal epithelial barrier integrity (24), and modulate T-cell differentiation by inhibiting histone deacetylases. Similarly, SBAs, exemplified by ursodeoxycholic acid, maintain immune balance via the activation of nuclear receptors such as the farnesoid X receptor (FXR) and Takeda G protein-coupled receptor 5 (TGR5), effectively restraining pro-inflammatory signaling in the liver (25–27).

A critical consequence of dysbiosis is overgrowth of Gram-negative bacteria, which elevates LPS production (28, 29). Disruption of intestinal barrier integrity due to dysbiosis further facilitates translocation of LPS into enterohepatic circulation (**Figures 1B, C**). Upon reaching the liver, LPS activates KCs predominantly via the Toll-like receptor 4 (TLR4) pathway, triggering pro-inflammatory cytokine cascades (**Figure 1C**). This mechanism not only amplifies hepatic inflammation, but also exacerbates disruption of intestinal barrier proteins, creating a cycle of gut barrier dysfunction, systemic endotoxemia, and chronic liver injury (30). Clinical observations reinforce this link, as elevated circulating LPS and soluble CD14 levels correlate with poorer outcomes in AIH patients (31).

Beyond LPS-induced inflammation, gut microbiota-derived metabolites engage additional signaling pathways implicated in IMLDs. SCFAs may influence hepatic immunity partly via stimulator of interferon genes (STING) activation, as evidenced by studies in intestinal models where STING modulates acetate-producing flora to regulate IgA production (32). Although direct roles of SCFAs in hepatic diseases require further elucidation, STING-mediated signaling—also triggered by LPS—plays an important role in hepatic inflammation and injury (33). Moreover, trimethylamine-N-oxide (TMAO), a metabolite derived from gut bacterial processing of choline, compromises intestinal barrier function and, via enterohepatic circulation, contributes to sinusoidal endothelial dysfunction, macrophage polarization, and indirect STING pathway activation, ultimately exacerbating hepatic fibrosis and inflammation (34). Additionally, gut-derived SBAs regulate immune responses primarily through FXR and TGR5 signaling. Activation of FXR restores intestinal barrier integrity, fine-tunes intestinal-vascular barrier permeability, and regulates bile acid synthesis via the ileal FGF15/19 feedback pathway. Dysbiosis disrupts this network, linking gut microbial imbalance to pathological processes ranging from inflammation to fibrosis and carcinogenesis in IMLDs (35).

The coordination of multiple signaling axes—such as SCFA–G-protein coupled receptor, LPS–TLR4, bile acid–FXR/TGR5, and STING pathways—demonstrates a complex, multi-layered mechanism through which gut microbiota metabolites modulate liver immunity (7, 36). Advanced mechanistic insights have highlighted these pathways as pivotal targets for developing microbiota-directed therapies to intervene in IMLDs.

### 2.2 Gut microbial translocation and immune trafficking

Gut microbiota dysbiosis has been proposed to contribute directly to PBC pathogenesis through the mechanism of molecular mimicry (37, 38). This concept is supported by the observed high sequence homology between the immunodominant epitope region of the human pyruvate dehydrogenase complex-E2 (PDC-E2) and its bacterial counterpart, particularly in *Escherichia coli* (37). Such homology suggests potential cross-reactivity, whereby an immune response initially directed against microbial PDC-E2 inadvertently triggers activation of autoreactive B and T cells targeting the human protein. Direct immunological evidence for this phenomenon stems from studies showing that sera from PBC patients simultaneously recognize both human and *E. coli*-derived PDC-E2 antigens (39). Beyond *E. coli*, other bacteria such as *Novosphingobium aromaticivorans* and *Lactobacillus delbrueckii* have also shown similar associations (40).

Leaky gut is a common consequence of dysbiosis, facilitating the translocation of microbial-associated molecular patterns (MAMPs), such as lipopolysaccharide (LPS), into the portal circulation. These MAMPs are recognized by innate immune cells in the liver, including KCs and hepatic stellate cells, via Toll-like receptors (TLRs), triggering the release of pro-inflammatory cytokines such as IL-1β, IL-6, and IL-23. For example, LPS, as a bacterial-derived hepatotoxic substance, induces immune responses in both the intestine and liver via the TLR4 or TLR4-NF-κB signaling pathway triggered by intestinal barrier disruption, ultimately contributing to the development of AIH (41). Notably, this cytokine combination (IL-1β, IL-6, and IL-23) is pivotal in driving the differentiation and expansion of naïve CD4^+^ T cells into T helper 17 (Th17) cells. Emerging research evidence indicates that gut microbiota dysbiosis in AIH and PBC significantly impacts the quantity and function of immune cell populations, including IL-17A-expressing CD4^+^ T cells, by altering harmful bacteria and microbial metabolite profiles (e.g., tryptophan derivatives and SBAs), disrupting immune homeostasis (42, 43). Kunzmann et al. demonstrated a significant increase in IL-17A-producing CD4^+^ T cells in PSC patients compared to healthy controls (44). Stimulation with *Candida albicans* and *Enterococcus faecalis*—microorganisms potentially translocated due to compromised intestinal barrier integrity—led to elevated IL-1β/IL-6 secretion by PSC monocytes, further promoting Th17 cell differentiation. These findings collectively position Th17 cell differentiation as a functional bridge between pro-inflammatory microbiota and T cells in the pathogenesis of the immune−mediated liver diseases, warranting further investigation as a potential therapeutic target (**Figure 1D**).

### 2.3 Gut microbiota dysbiosis and its association with the pathogenic mechanisms of AIH

Dysbiosis disrupts this equilibrium through several key mechanisms, profoundly influencing AIH progression (45). Metabolites derived from specific bacterial strains demonstrate direct hepatotoxicity—for example, cytolysin secreted by *Enterococcus faecalis* triggers hepatocyte apoptosis (46), while ethanol produced by the same bacterium exacerbates injury via oxidative stress mechanisms (47). More critically, gut microbiota dysbiosis-induced bile acid metabolic abnormalities disrupt immune balance via dual receptor mechanisms: reduced levels of SBAs (DCA, LCA) lead to diminished FXR signaling pathway activity (48), impairing regulatory T cell (Treg) function, while insufficient TGR5 activation promotes Th17 cell differentiation, ultimately breaking hepatic tolerance to commensal antigens (49) (**Figure 1E**).

The association between gut microbiota dysbiosis and AIH has been confirmed by multiple studies, with research across different regions demonstrating significant correlations between microbial signatures (50). In eastern China, AIH patients exhibit significantly reduced gut microbiota diversity, with increased abundance of pathogenic genera such as *Veillonella* and *Klebsiella* (31, 51). More remarkably, a study in central China identified five key genera (*Lachnospiraceae*, *Veillonella*, *etc.*) as biomarkers through multi-omics analysis, further improving diagnostic accuracy to 83.25% (52). These regional variations in microbial composition highlight the importance of considering geographical factors when investigating the gut-liver axis in AIH pathogenesis. However, the observed geographical differences in gut microbiota profiles among AIH patients may be influenced by several confounding factors that warrant careful consideration. Dietary patterns represent a major contributor to microbiome variation, as different populations consume distinct food compositions that shape microbial communities. For instance, the traditional Chinese diet, characterized by high fiber intake and fermented food consumption, may produce different microbial metabolites compared to Western dietary patterns (53). Additionally, treatment status significantly impacts microbiota composition, as immunosuppressive therapies and corticosteroid use can alter microbial diversity and functional pathways (54, 55). These factors collectively contribute to the complexity of interpreting microbiota data and underscore the necessity for standardized protocols and multi-center collaborative studies to establish robust, reproducible findings.

The nature of microbiome changes in AIH remains a subject of debate. Some studies indicate that certain microbial alterations, such as reductions in *Bifidobacterium* and *Coprococcus* and an expansion of *Streptococcus*, persist after therapy (56, 57). However, it remains unclear whether these changes represent compensatory effects or primary pathogenic features. Another significant gap in the literature is the inconsistency in proposed microbial biomarkers across different geographical populations. For example, signature taxa identified in eastern China (e.g., *Veillonella*, *Lactobacillus*) differ from those highlighted in central China (e.g., *Lachnospiraceae*) (51, 58). These regional discrepancies in microbial signatures reflect the presence of contradictory research findings across studies, likely influenced by variations in host genetics, diet, and environmental exposures. The lack of standardized profiling methods further complicates cross-study comparisons and challenges the identification of universal disease biomarkers.

Current research on the association between gut microbiota and AIH presents confusing findings: some studies report a significant correlation between increased abundance of specific bacterial taxa (e.g., *Enterococcus faecalis and Enterococcus gallinarum*) and AIH disease activity (59), while *Enterococcus* Capsules observe inconsistent or even contradictory associations across in the treatment of AIH mice (60). These discrepancies may stem from variations in demographic characteristics, diagnostic criteria, or methodological approaches (e.g., 16S rRNA sequencing versus shotgun metagenomic sequencing). At the molecular level, antigenic similarity between gut microbiota and hepatocyte surfaces [*e.g.*, epitope overlap between *Escherichia coli* (*E. coli*) pyruvate dehydrogenase complex E2 subunit (PDC-E2) and hepatocyte antigens] may trigger autoimmune responses through molecular mimicry (61). Recent findings also highlight the depletion of SCFAs-producing bacteria (*e.g.*, *Faecalibacterium prausnitzii*) in AIH patients, resulting in reduced anti-inflammatory metabolites and exacerbated hepatic inflammation (**Figure 1C**) (62).

The mechanistic links described above are supported by a combination of animal models and clinical observations. Key signaling (e.g., FXR/TGR5, TLR4-MyD88) have been validated in rodent studies, which offer controlled systems for mechanistic dissection but are limited by interspecies differences. A key unresolved question is whether gut dysbiosis initiates AIH or is a secondary consequence of chronic liver inflammation (59). Therapeutically, interventions targeting the gut microbiota have shown promising clinical potential: specific probiotic formulations can significantly reduce serum ALT levels (63), and fecal microbiota transplantation (FMT), by restoring the “microbiota-bile acid-immune” axis, has achieved biochemical remission in refractory cases of AIH (63, 64). However, the therapeutic efficacy of FMT in AIH requires further validation through additional clinical studies.

### 2.4 Gut microbiota dysbiosis and its association with the pathogenic mechanisms of PBC

PBC, an autoimmune liver disorder predominantly affecting middle-aged women, is characterized by anti-mitochondrial antibody (AMA) positivity and progressive destruction of the small intrahepatic bile ducts, potentially leading to cholestatic cirrhosis and eventual liver failure (65). Clinical epidemiological data indicate that PBC exhibits an age-dependent incidence, with the highest disease-specific burden concentrated in the 60–79 age group (66). Despite the established use of ursodeoxycholic acid (UDCA) and obeticholic acid (OCA) as standard therapeutic agents, nearly 40% of patients demonstrate suboptimal biochemical response, a clinical challenge that has directed research attention toward the gut-liver axis for deeper mechanistic insights (67, 68).

A consistent finding across studies is the marked alteration in gut microbial composition observed in PBC patients. These changes are characterized by a reduced richness and diversity, an expansion of pro-inflammatory genera such as *Haemophilus* and *Veillonella* (69), and a relative paucity of anti-inflammatory, SCFA-producing taxa including *Oscillospira* and *Faecalibacterium* (70). Notably, the depletion of specific bacterial clades with known bile acid decoupling capabilities – notably *Actinobacteria* and *Desulfovibrio* – has been linked to disturbances in bile acid homeostasis. This could result in both compositional abnormalities and the toxic accumulation of SBAs, a condition that may further impair the therapeutic efficacy of UDCA (67, 68). The resulting disturbance establishes a bidirectional regulatory loop between the gut and liver, primarily mediated through the modulation of bile acid metabolism, the disruption of intestinal barrier integrity, and the subsequent activation of both innate and adaptive immune pathways (70).

Experimental evidence from animal models further underscores the pathogenic potential of microbiota alterations. In the NOD.c3c4 mouse model – a model of autoimmune-mediated cholangitis – studies demonstrated that germ - free conditions correlated with attenuated liver disease severity when compared to conventionally raised counterparts. Antibiotic intervention was shown to significantly ameliorate T cell-mediated cholangitis in these animals (70). These lines of evidence highlight that gut microbiota alterations constitute a critical etiological factor in PBC pathogenesis, capable of initiating autoimmune attacks against bile duct epithelium primarily through the disruption of peripheral immune tolerance mechanisms (**Figure 1E**). **Figure 1** provides a visual summary of the proposed mechanistic cascade linking dysbiosis to PBC.

The pathogenic mechanisms of gut microbiota dysbiosis in inducing PBC, as outlined above, highlight unresolved questions regarding the translational validity of findings from PBC animal models to human disease. These include the relative contributions of specific microbial species (e.g., *E. coli* versus *Novosphingobium*), the precise molecular pathways involved, and the potential clinical utility of targeted interventions—such as probiotics, prebiotics, or fecal microbiota transplantation—which require rigorous clinical trials for validation (71).

The evidence linking the gut microbiota to PBC is derived from both clinical observational studies and experimental models, yet it is constrained by several methodological limitations that necessitate cautious interpretation. Clinical correlations predominantly stem from case-control studies with limited sample sizes, which are inherently susceptible to confounding variables, including dietary habits, concurrent medications (*e.g.*, antibiotics and UDCA), and geographical heterogeneity. Despite molecular mimicry being a plausible pathogenic mechanism, its universal occurrence remains unconfirmed. Key methodological weaknesses in the existing literature include: (1) the predominance of case-control studies with limited sample sizes in clinical research, which are inherently vulnerable to confounding variables such as diet, concurrent medications, and geographical heterogeneity; (2) the cross-sectional design of most human studies, which precludes the establishment of temporal relationships and makes it challenging to determine whether observed microbial alterations are a cause or consequence of PBC; and (3) technical variations in sampling methods (*e.g.*, stool vs. mucosal biopsies), DNA extraction protocols, and sequencing platforms (e.g., 16S rRNA vs. shotgun metagenomics), which introduce substantial inter-study variability, thereby limiting reproducibility and the integration of findings through meta-analysis.

### 2.5 Gut microbiota dysbiosis and its association with the pathogenic mechanisms of PSC

PSC constitutes a chronic cholestatic liver disease with distinct epidemiological characteristics (72), including a global incidence of 0.9–1.3 cases per 100,000 individuals and significantly higher prevalence in European and American populations (3.85–16.2/100,000) compared to Asian regions (73, 74). The condition exhibits a male predominance with a sex ratio of approximately 2:1–3:2 and features a bimodal age distribution, with incidence peaks around 15 and 35 years of age (75). The strong clinical association between PSC and IBD, affecting around 80% of patients, underscores the central role of gut–liver axis dysfunction in disease pathogenesis (**Figure 1D**) (75, 76). Patients also contend with substantially elevated risks of cholangiocarcinoma and colorectal cancer, presenting persistent clinical management challenges (77, 78).

Alterations in gut microbiota composition significantly contribute to PSC pathogenesis through interconnected biological mechanisms involving structural compromise, metabolic dysfunction, and immune dysregulation (79). PSC patients exhibit notably reduced gut microbial diversity, with characteristic alterations including expansion of pro-inflammatory bacterial taxa such as *Enterobacteriaceae*, *Veillonella*, and *Streptococcus* (80, 81), alongside depletion of beneficial short-chain fatty acid–producing bacteria like *Faecalibacterium* (82). The administration of vancomycin in mice results in greater hepatic collagen deposition and injury, due to the blockage of the intestinal FXR-FGF15/19 axis and elevated bile acid levels. These impacts are linked to the decrease in *Clostridia XIVa*, particularly *Clostridium scindens*. Administering *Clostridium scindens* orally reduces vancomycin-induced bile acid buildup and liver fibrosis by activating intestinal FXR-FGF15/19 signaling. Mice receiving engineered Escherichia coli Nissle 1917, which can express bile acid 7α-dehydratas (BaiE) from Clostridium scindens (EcN-BaiE), exhibit similar effects (83). Fungal communities similarly display dysbiosis, marked by enrichment of opportunistic pathogens including *Exophiala* and *Candida albicans*, and reduction of potentially protective species such as *Saccharomyces cerevisiae* (84, 85) (**Figure 1E**).

Metabolic disturbances, particularly in SBAs processing, foster inflammatory cell recruitment around bile ducts and disrupt bile acid and gut microbiota homeostasis, driving immune activation and fibrotic progression in PSC. These disrupted interactions may compromise the intestinal epithelial barrier, allowing bacterial translocation and promoting systemic inflammation. A critical but unresolved question is whether gut dysbiosis represents a primary causative factor or a secondary consequence in PSC pathogenesis. The precise molecular mechanisms linking specific microbial taxa (e.g., *Veillonella* or *Candida albicans*) to selective bile duct inflammation remain incompletely understood (86). Moreover, it is unclear whether interventions such as FMT or targeted probiotics that aim to restore specific microbial taxa or their metabolites can significantly modify the natural history of the disease in PSC patients.

Nakamoto’s team identified *Klebsiella pneumoniae* (*K. pneumoniae*) in the gut microbiota of PSC patients (87). Mouse models revealed that this bacterium induces pore formation in human intestinal epithelial cells, disrupting barrier function and causing dysbiosis. FMT from PSC patients to germ-free (GF) mice successfully isolated *K. pneumoniae*, *Proteus mirabilis*, and *Enterococcus faecalis* from mesenteric lymph nodes. These three bacteria collaboratively promote hepatobiliary disease progression via Th17 immune responses, with *K. pneumoniae* playing a pivotal role in activating liver Th17 responses mediated by intestinal epithelial damage (**Table 1**).

**Table 1:** Key molecular mechanisms of gut microbiota dysbiosis in IMLDs.

Category	Names	Resource	Receptors or main mechanism	Effects	The immune−mediated liver diseases	References
Microbiota-derived metabolites	BAs	Microbial metabolism (secondary BAs, isoallo-LCA, etc.)	FXR/TGR5 pathway	Regulating liver BA synthesis	PSC, PBC	(88)
	SCFAs	Anaerobic fermentation of indigestible proteins and fibers.	GPR41, GPR 43, GPR109a or promote Treg differentiation and inhibit NF-κB pathway	Cellular energy supply; Anti-inflammation.	AIH	(89)
	Tryptophan and indols	Foods like vegetables, fish, and eggs, *etc.*	AhR signaling pathway activation in T cells	Host metabolizing via the kynurenine and serotonin pathway; Anti-inflammation via indole and indole-related derivatives	AIH	(90, 91)
Gut microbial translocation	Microbiota-derived hepatotoxins	Bacterial LPS	TLR4 or TLR4-NF-κB	Triggering gut immune response; Arriving liver via portal vein and launching liver immune response.	AIH	(41, 92)
	Gut microbes	Bacterial LPS and alive gut microbes	Trigger Th17 immune response	Trigger an immune attack against their own PDC-E2.	PBC	(37)
Gut-liver immune trafficking	Microbes-activated immune cells	Gut immune cells		A rolling interaction mediated by selectins, integrins; Trafficking into the liver and mediating immune response.	AIH, PBC, PSC	(43, 90, 93–95)

IMLDs, immune-mediated liver diseases; isoallo-LCA, isoallolithocholic acid; BAs, bile acids; FXR, farmesoid X receptor; GPR, G-protein coupled receptor; LPS, lpopolysaccharides; PBC, primary biliary cholangiti; PSC, primary sclerosing cholangiti; SCFAs, Short chain fatty acids. Same color indicates the same type of mechanism characteristics.

Fungal metabolites may also translocate to the liver during barrier dysfunction. *Candida albicans* secretes peptide toxin Candidalysin, which directly induces hepatocyte death by activating MAPK/c-Fos/MAP kinase phosphatase-1 signaling pathways (96). Additionally, specific gut fungi can signal liver abnormalities via antigen-reactive cells (including Th17 cells) (97). Clinical evidence shows that PSC patients with biliary candidiasis exhibit more severe cholangitis, elevated C-reactive protein, and hyperbilirubinemia, absent in non-candidiasis cases (98). While fungal contributions to autoimmune liver disease pathogenesis remain hypothetical, these findings collectively support a conceptual framework: in genetically susceptible hosts, fungal components—by activating innate immune receptors and modulating the Th17 axis—may amplify PSC. Future studies are needed to validate this hypothesis in autoimmune contexts.

The current body of evidence on this topic exhibits several limitations. Epidemiological data predominantly originate from retrospective cohort studies conducted in high-incidence regions, with limited representation of Asian populations. Many reported microbiota alterations are based on case-control studies with restricted sample sizes, which may introduce potential confounders such as concomitant inflammatory bowel disease (IBD) and prior antibiotic exposure (99). While animal models (e.g., *Mdr2^–/–^* mice) provide some support for causality, they do not fully replicate the complex interaction between PSC and IBD observed in humans, nor do they adequately model the prolonged, chronic evolution of the disease (100).

The current evidence on this topic has certain limitations. Furthermore, significant translational barriers exist: (1) Animal models (e.g., Mdr2^–/–^ mice) do not fully replicate the complex human disease phenotype, particularly the strong association with IBD observed in 80% of PSC patients; (2) Most preclinical studies utilize acute injury models that fail to capture the chronic, progressive nature of human PSC spanning decades; (3) Interspecies differences in bile acid composition, immune system architecture, and gut microbiota ecology limit the direct applicability of mechanistic insights from mice to human therapeutic development; (4) The lack of validated surrogate endpoints in clinical trials (beyond alkaline phosphatase reduction) hinders the efficient evaluation of novel microbiota-targeting interventions.

## 3 Therapeutic strategies

Gut microbiota dysbiosis actively contributes to the pathogenesis of IMLDs. This involvement is mediated through several interconnected mechanisms: alterations in microbial metabolism (e.g., abnormal profiles of SCFAs and SBAs) and the consequent dysregulation of key immune signaling pathways (e.g., TLR4/NF-κB, FXR/TGR5). In response, emerging therapeutic approaches are increasingly focused on targeting this microbiota-immune network. Strategies such as bile acid receptor agonism and FMT have gained significant research momentum as promising interventions.

### 3.1 SBAs as diagnostic biomarkers

Under normal gut microbiota homeostasis, gut bacteria convert primary bile acids into secondary bile acids (SBAs) through enzymatic reactions, including bile salt hydrolysis by bile salt hydrolase (BSH) and 7α-dehydroxylation to form deoxycholic acid (DCA) and lithocholic acid (LCA). Gut dysbiosis alters the abundance and activity of bacteria responsible for producing these enzymes, leading to aberrant bile acid transformation, significantly altering the generation and composition of SBAs (101). These compounds, such as deoxycholic acid (DCA) and lithocholic acid (LCA), are primarily synthesized by gut bacteria through 7α-dehydroxylation of primary bile acids (102). Dysbiosis leads to reduced abundance of key bacterial species (e.g., *Clostridium* species), thereby decreasing SBAs production (103). For example, the reduction of *Bacteroides uniformis* and its metabolic product 3-succinylcholic acid (3-sucCA) has been shown to significantly increase the risk of liver inflammation in murine models (104). The reduction of SBAs has profound implications for immune regulation. As endogenous ligands of the FXR/TGR5 signaling pathways, decreased SBAs weaken receptor activation, compromise intestinal barrier integrity, and exacerbate inflammatory responses (105). Clinical studies demonstrate that bile acid metabolism disruptions induced by dysbiosis increase intestinal permeability, promoting bacterial translocation and immune dysregulation. This phenomenon establishes a “metabolic-inflammatory” vicious cycle, wherein gut dysbiosis drives hepatic inflammation through impaired bile acid metabolism.

Other microbiota-derived SBAs, such as isoallolithocholic acid (isoallo-LCA), exhibit significant accumulation in IMLDs, showing strong correlation with clinical disease activity (106). This positions them as potential molecular tools for early diagnosis and disease monitoring. As terminal metabolites of host cholesterol processed by gut bacteria, their abnormal levels serve as direct indicators of intestinal dysbiosis and can influence hepatic energy metabolism and immune regulation via activation of hepatic receptors like FXR and TGR5 (105, 107). Clinical studies further substantiate that serum isoallo-LCA concentrations are positively correlated with elevated serum ALT and AST levels in patients with hepatic dysfunction (108). Notably, the ratio of LCA to ursodeoxycholic acid (UDCA) has been shown to effectively discriminate between compensated and decompensated cirrhosis, offering a novel tool for precise disease staging. These insights not only illuminate the centrality of the gut microbiota–bile acid axis in liver pathophysiology but also establish a theoretical foundation for developing personalized therapeutic strategies based on bile acid modulation.

Translating these mechanistic insights into therapy, the synthetic FXR agonist obeticholic acid has demonstrated multifaceted efficacy in preclinical models (109, 110). Its action, through specific activation of bile acid receptor signaling, results in amelioration of hepatic inflammation, improvement of metabolic parameters, enhancement of intestinal barrier integrity, and favorable modulation of gut microbiota composition (109, 111). This coordinated, multi-tissue effect underscores the therapeutic importance of FXR activation in conditions characterized by impaired bile acid signaling, such as IBD and non-alcoholic fatty liver disease (112).

### 3.2 FMT

FMT seeks to restore the dynamic equilibrium of gut microbiota, thereby rebalancing the metabolite environment that shapes hepatic immunity. In experimental settings, transplanting fecal microbiota from PBC patients into pseudo-germ-free mice (PBC-FMT) elevated serum alkaline phosphatase (ALP) and total bile acid levels, induced liver injury, and enriched hepatic gene pathways linked to disease (113). The PBC-FMT group showed upregulated immune and signaling pathways alongside downregulated metabolic pathways, reflecting molecular changes observed in human PBC. Similarly, in a 2-OA-BSA-induced PBC model, PBC-FMT worsened liver damage, indicating a key pathogenic role of gut dysbiosis (113). These findings highlight microbiota-targeted strategies, especially FMT, as a promising therapeutic direction for PBC.

Supporting this, a case report noted that FMT markedly improved liver function, bile acid profiles, and bacterial communities in a PSC patient with recurrent bacterial cholangitis (114). In cirrhosis patients—often marked by dysbiosis and immune dysfunction—FMT has been shown to safely restore microbial balance, lower hospitalization rates, improve cognitive function, and reduce hepatic encephalopathy incidence (115). Clinical trials also report FMT’s effectiveness in reducing hepatic fat accumulation by mitigating gut dysbiosis and strengthening intestinal barrier function (116, 117).

In an AIH mouse model, therapeutic FMT using fresh feces from healthy mice reversed liver injury through the TLR4-MyD88 signaling pathway (63). The first dedicated clinical trial in PSC patients demonstrated favorable safety of FMT, along with a significant and sustained increase in overall gut microbial diversity post-treatment. Moreover, a positive correlation was found between the abundance of engrafted operational taxonomic units (OTUs) and decreased serum ALP levels, suggesting a link between microbial restoration and improved hepatic biochemistry (118).

Collectively, these studies highlight the therapeutic potential of FMT across a spectrum of liver diseases. An important advancement in FMT methodology involves the remodeling of recipient gut ecology and restoration of bile acid metabolic homeostasis through the transfer of functional microbiota from healthy donors (119, 120). Clinical evidence indicates that FMT not only enhances beneficial bile acid production but also ameliorates dysbiosis-related pathology via activation of bile acid receptors. In a phase I trial by Allegretti et al., PSC patients with concomitant IBD who received FMT over six weeks showed significantly increased gut microbiota alpha diversity, with 30% of participants achieving a >50% reduction in ALP levels without serious adverse events (118).

**Figure 2:**
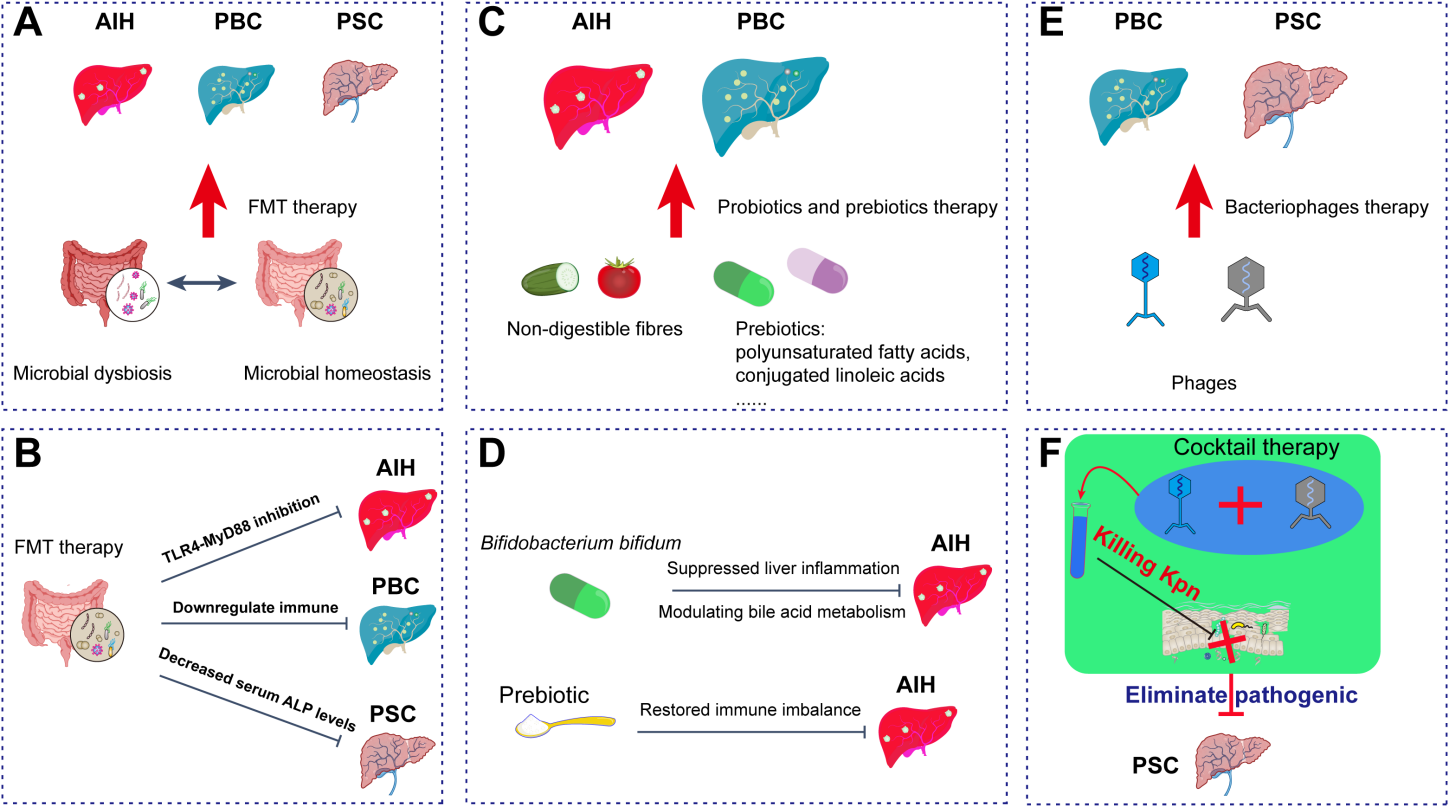
Gut microbiota-derived main therapeutic strategies for IMLDs. **(A)** Potential therapeutic applications of FMT in AIH and PBC; **(B)** FMT downregulates immune responses through TLR4−MyD88 inhibition and decreases serum ALP levels; **(C)** Potential therapeutic effects of probiotics and prebiotics in AIH and PBC; **(D)** Prebiotics and *Bifidobacterium bifidum* alleviate hepatic inflammation in AIH by modulating bile acid metabolism and restoring immune balance; **(E)** Potential therapeutic role of bacteriophages in PBC and PSC; **(F)** Bacteriophage cocktail therapy selectively eliminates PSC−associated pathogenic bacteria (*e.g.*, Kpn). FMT, fecal microbiota transplantation; AIH, autoimmune hepatitis; PBC, primary biliary cholangitis; PSC, primary sclerosing cholangitis. Kpn, *Klebsiella pneumoniae*.

Despite these promising results, adherence to strict international guidelines for donor screening and standardized fecal banking remains crucial to address challenges related to treatment durability and long-term safety (**Figures 2A, B**).

### 3.3 Probiotics and prebiotics

In AIH models, supplementation with *Bifidobacterium* animalis subsp. *lactis* 420 significantly reduced serum endotoxin levels and suppressed the activation of the receptor-interacting protein kinase 3 (RIP3) signaling pathway in liver macrophages. This effect was accompanied by elevated fecal SCFA levels and upregulation of tight junction protein expression (115). In an LPS-induced liver inflammation model, administration of *Bifidobacterium bifidum* markedly enhanced hepatocyte metabolic activity via gut-liver axis mechanisms (6, 121), indicating its potential as a microbiome-targeted strategy for managing hepatic inflammation. Beyond conventional probiotics, *Akkermansia muciniphila* has emerged as a promising beneficial microorganism due to its multifaceted roles in maintaining microbial balance, strengthening the intestinal barrier, modulating bile acid metabolism, and regulating host immune responses (122).

Prebiotics, though indigestible by the host, enhance probiotic activity through microbial fermentation, thereby conferring health benefits (123). For example, C57BL/6 mice were administered prebiotics for 7 consecutive days, followed by induction of an AIH model via tail vein injection of concanavalin A. Prebiotic intervention was found to reduce the levels of pro-inflammatory cytokines, increase the concentrations of anti-inflammatory factors, and correct the immune imbalance in AIH. Furthermore, this intervention could reshape the disrupted gut microbiota, maintain intestinal barrier integrity, and block the activation of the LPS/TLR4/NF-κB pathway in the liver (95). *Eurotium cristatum*-fermented dark tea increases the relative abundance of beneficial genera such as *Lactobacillus*, *Akkermansia*, and *Faecalibacterium*, while raising SCFA levels, synergistically inhibiting liver inflammation and oxidative stress (124). Similarly, fermented quinoa and black barley elevate the abundance of probiotic taxa and upregulate microbial bile acid synthesis pathways, collectively alleviating chronic liver inflammation induced by a high-fat diet (125).

Prebiotic compounds encompass conjugated linoleic acid, polyunsaturated fatty acids, and various oligosaccharides (126). Unlike ordinary dietary fibers, prebiotics selectively stimulate the metabolic activity of beneficial host microorganisms (127). Through fermentation, they modulate the composition of the gut microbiota and provide energy for host cells. Short-chain fatty acids, as key fermentation products, play a significant anti-inflammatory role by regulating immune responses and coordinating biological functions (128).

Collectively, these findings highlight the central role of the gut microbiota–immune axis in maintaining homeostasis in autoimmune hepatitis (AIH). While current research on prebiotics and their compounds in PBC and PSC remains limited, existing evidence offers new perspectives for the precision application of prebiotics in the treatment of immune-mediated AIH (**Figures 2C, D**).

### 3.4 Bacteriophages therapy: a novel intervention

As an innovative and precise intervention, bacteriophage (phage) therapy offers a novel avenue for treating IMLDs by virtue of its specific bacterial targeting capability. These natural viruses can selectively eliminate pathogenic gut bacteria through specific receptor binding while preserving the broader microbial ecosystem’s stability (129). For instance, in PSC, researchers have developed a lytic phage cocktail demonstrating sustained inhibitory effects against PSC-associated *Klebsiella pneumoniae* (Kpn) *in vitro*. Animal studies confirm that oral administration of this cocktail significantly reduces Kpn colonization in GF and specific pathogen-free mice without causing off-target dysbiosis, supporting its clinical translational potential (130).

In the broader context of IMLDs, phage therapy exhibits multi-dimensional application potential. A combinatorial immunoglobulin library constructed using a λ phage vector system enables the isolation of high-affinity Fab fragments targeting specific autoantigens, serving as a valuable tool for investigating PBC immune mechanisms (131). In alcoholic hepatitis, where approximately 80% of patients harbor cytolysin-producing *Enterococcus faecalis*, targeted phage therapy completely eliminated the liver injury phenotype in GF mice. For NAFLD associated with high-alcohol-producing *Klebsiella pneumoniae* (HiAlc Kpn), phage therapy can specifically clear the pathogen, alleviating steatohepatitis (132). Notably, engineered phages have been explored for viral hepatitis, with surface-displayed hepatitis B core antigens (HBcAg) inducing neutralizing antibodies, suggesting novel therapeutic strategies (129). For immunocompromised patients with cirrhosis or liver transplants, phage therapy can also effectively prevent drug-resistant infections (133, 134). These advances validate the pivotal role of phages in regulating the gut-liver axis and highlight their value as precision tools—by selectively eliminating pathogens, restoring microbial balance, and blocking upstream immune activation to provide personalized treatment options (**Figures 2E, F**).

### 3.5 Translational challenges and barriers

Despite promising preclinical evidence, several critical barriers impede the clinical translation of gut microbiota-targeted therapies for IMLDs. First, methodological standardization remains inadequate – variations in FMT preparation protocols, probiotic strain selection, and dosing regimens across studies create significant reproducibility issues. Second, patient stratification complexity presents a major hurdle, as individual variations in baseline microbiota composition, host genetics, disease stage, and concurrent medications profoundly influence therapeutic responses. Third, regulatory pathways for microbiome-based therapies are underdeveloped, particularly for phage therapy and engineered probiotic products, creating uncertainty in clinical development pipelines. Fourth, long-term safety concerns persist, especially regarding the potential for FMT to transmit unrecognized pathogens or trigger unexpected immune reactions in immunocompromised liver disease patients. Finally, economic and accessibility barriers may limit the implementation of personalized microbiota therapies, particularly in resource-limited settings where IMLDs are increasingly prevalent.

## 4 Current status and stratified clinical insights

Among these strategies, FMT currently possesses the most clinical validation, supported by positive outcomes in early-phase trials for conditions like PSC−IBD overlap syndrome. Bile acid receptor agonists are underpinned by robust mechanistic data from related liver diseases. Probiotics and prebiotics primarily rely on supportive evidence from animal models and small-scale human studies, while bacteriophage therapy remains at a compelling preclinical proof-of-concept stage, lacking registered clinical trials in liver diseases. Safety profiles vary: FMT is generally safe in regulated settings but requires stringent donor screening; probiotics have favorable safety profiles but may cause transient ecological disruption; phage therapy demonstrates high specificity with minimal off-target effects in models.

The applicability of these interventions is stage-dependent. Probiotics/prebiotics and bile acid modulators may offer preventive benefits in early-stage disease, while FMT shows particular promise in established conditions like PSC−IBD or cirrhosis with recurrent encephalopathy. Phage therapy is considered an investigational option for refractory, pathogen-driven phenotypes. Significant challenges persist: FMT faces issues with engraftment durability and protocol standardization; phage therapy contends with rapid resistance emergence and regulatory hurdles; probiotic formulations suffer from inconsistent viability and strain-specific effects; and bile acid therapeutics may have reduced efficacy in advanced fibrosis. These stratified insights assist clinicians in aligning intervention profiles with individual patient characteristics—for example, prioritizing FMT in advanced PSC−IBD, reserving phage therapy for investigational use in resistant infections, and employing probiotics as adjunctive modulators alongside conventional immunosuppression.

## 5 Summary and prospects

This review provides a comprehensive analysis of the critical role gut microbiota dysbiosis plays in the pathogenesis of immune-mediated liver diseases, including AIH, PBC, and PSC. Accumulating evidence confirms that gut microbes influence disease progression through diverse mechanisms, such as generating bioactive metabolites (e.g., SCFAs, SBAs), modulating immune pathways (e.g., TLR4/NF-κB, FXR/TGR5 signaling cascades), and participating in molecular mimicry events. Clinically, the intricate gut-liver axis connection is underscored by observations that 80% of PSC patients present with concurrent IBD, and the annual risk of cholangiocarcinoma in these individuals is 1–3%. Dysbiosis is often characterized by the proliferation of pro-inflammatory bacterial taxa (e.g., *Enterobacteriaceae*) and a concomitant depletion of protective commensals (e.g., *Akkermansia*), a perturbation that can compromise intestinal barrier integrity, disrupt bile acid metabolism, and drive aberrant T helper 17 (Th17) immune responses.

This review makes two key innovative contributions beyond existing research. First, it integrates common mechanisms with disease-specific differences, demonstrating that gut microbiota dysbiosis drives IMLDs through shared pathways like bile acid metabolism disorders and TLR4 activation, while highlighting distinct pathological features in diseases such as AIH and PBC, providing a systematic framework for understanding IMLD heterogeneity. Second, it reveals microbial metabolites—including SBAs, SCFAs, and tryptophan derivatives—as novel “molecular bridges” linking gut dysbiosis to hepatic inflammation, serving as key mediators by modulating FXR, TGR5, and STING signaling pathways, thereby offering a groundbreaking, integrative perspective for mechanistic research in this field.

Current therapeutic strategies leveraging the gut-liver axis demonstrate tangible, albeit variable, clinical promise. For example, FMT has been shown to induce a greater than 50% reduction in alkaline phosphatase levels in approximately 30% of PSC patients (118). Similarly, targeted phage therapy offers a precise means of eliminating pathogenic strains (e.g., cytolysin-producing *Enterococcus faecalis*), while obeticholic acid enhances immune tolerance by modulating the FXR signaling pathway.

To translate these preliminary findings into robust clinical applications, future investigations should concentrate on three key fronts. First, mechanistic depth must be enhanced through advanced single-cell sequencing and metabolomic platforms to delineate the dynamic regulatory functions of gut-derived factors in bile duct repair and fibrogenesis. Second, technological innovation should be prioritized to develop next-generation delivery systems, such as CRISPR-engineered probiotic vectors, personalized combinatorial regimens (e.g., OCA with specific probiotic strains like *Akkermansia*), and optimized nano-probiotic complexes (e.g., BL@TA-FeIII@AgNPs) for precise therapeutic targeting. Third, rigorous efforts in clinical translation are imperative, including the establishment of international standards for donor screening in microbiota-based therapies, overcoming the challenge of low strain colonization efficiency, validating long-term safety, and testing emerging hypotheses, such as the role of fungal components (e.g., *Candida albicans*) in amplifying autoimmunity via the Th17 axis. Collectively, these multi-directional efforts will yield essential theoretical and practical frameworks to advance the precision prevention and management of IMLDs.

However, several limitations must be acknowledged in the current review. The primary constraint lies in the inherent differences between animal models and human physiology, particularly regarding immune system architecture and microbiome composition, which may significantly limit the translational relevance of findings from preclinical studies. Additionally, most human cohort studies to date have employed cross-sectional designs, making it difficult to establish temporal sequences or infer causality between gut microbiota alterations and disease progression. These methodological limitations underscore the critical need for prospective longitudinal studies that can validate observed associations and elucidate the causal relationships underlying gut-liver axis dysfunction in IMLDs. Furthermore, the heterogeneity of patient populations and the complexity of microbial interactions present additional challenges that require more sophisticated analytical approaches and larger-scale clinical investigations to fully understand the mechanistic pathways involved.
